# The Association of Sperm DNA Fragmentation With Serum Lipid Profile Among Males With Abnormal Semen Parameters: An Analytical Cross-Sectional Study

**DOI:** 10.7759/cureus.60243

**Published:** 2024-05-13

**Authors:** Raju Kumaran Thankamoni, Dharmaraj W Tamgire, Papa Dasari, Prashant S Adole, Sreerekha Jinkala, Kubera NS, Viveka Murugiah

**Affiliations:** 1 Anatomy, Velammal Medical College Hospital and Research Institute, Madurai, IND; 2 Anatomy, Jawaharlal Institute of Postgraduate Medical Education and Research, Puducherry, IND; 3 Obstetrics and Gynaecology, Jawaharlal Institute of Postgraduate Medical Education and Research, Puducherry, IND; 4 Biochemistry, Jawaharlal Institute of Postgraduate Medical Education and Research, Puducherry, IND; 5 Pathology, Jawaharlal Institute of Postgraduate Medical Education and Research, Puducherry, IND; 6 Biochemistry, Velammal Medical College Hospital and Research Institute, Madurai, IND

**Keywords:** serum lipid profile, infertility, semen analysis, comet assay, sperm dna fragmentation

## Abstract

Background: Through the ages, infertility, affecting 8% to 12% of couples worldwide, has been a perturbing clinical problem. Approximately 40% to 50% of all infertility cases are due to 'male factor' infertility. Semen analysis is crucial in routinely evaluating idiopathic male infertility. Studies support the idea that semen parameters are associated with serum lipids and sperm DNA fragmentation (SDF). Therefore, it is possible to evaluate male infertility by serum lipid levels, especially before assisted reproduction technology, and modify it by bringing about lifestyle modifications. This study aimed to measure the correlation of SDF with levels of total cholesterol (TC), triglycerides (TG), very low-density lipoprotein (VLDL), low-density lipoprotein (LDL), and high-density lipoprotein (HDL) among males with abnormal semen parameters.

Methods: A cross-sectional analytical study was conducted in the infertility clinic of a tertiary care hospital. A total of 106 infertile males with abnormal semen analysis as per the WHO criteria (2010) were enrolled in the study. After routine semen analysis, SDF was studied using the comet assay. The serum fasting lipid profile was analyzed using the spectrophotometric kit in the autoanalyzer. The relationship of SDF with serum lipid profile parameters was analyzed.

Results: Out of 106 infertile men, 52% (n = 55) had severe SDF. A modest positive correlation was observed between SDF (percentage of DNA in comet tail) and serum lipid values (serum TG, serum LDL, and serum VLDL).

Conclusions: Our study is novel in its research on the correlation between SDF and serum lipid values. Based on the findings of our study, it can be concluded that a significant level of SDF was observed in men with high levels of serum TG, LDL, and VLDL. This provokes a potential relationship between sperm DNA integrity and serum lipid profile, which warrants further research.

## Introduction

Infertility is a disease of the reproductive system that is defined as the inability of a non-contracepting, sexually active couple to achieve pregnancy in one year [1]. The social stigma associated with it causes severe trauma for an individual. In recent years, there has been a surge in infertility in most parts of the world, affecting 8% to 12% of couples, of which the sole cause in approximately 40% to 50% of cases belongs to the male [2].

A decline in semen quality and sperm parameters has been observed in South India over the past 13 years [3]. A routine semen analysis assessing a decrease in semen quality and quantity does not always give a definite diagnosis; i.e., in 15% of male factor infertility cases, the results are normal [4]. Additionally, with intracytoplasmic sperm injection (ICSI), conventional sperm analysis has fallen out of favor. More attention is being given to sperm molecular architecture, as mammalian fertilization and subsequent embryo development depend in part on the inherent integrity of sperm DNA [5].

Studies have noted diverse observations exploring the link between sperm DNA fragmentation (SDF) and infertility due to male factors. Several types of lipids, different in structure and function, are present in the seminal plasma of humans [6]. Male infertility may be affected by metabolic abnormalities of lipids [7]. Increased production of reactive oxygen species (ROS) is caused by increased circulating lipids, which provide energy substrates in adipose and non-adipose tissue towards its metabolic pathway. In approximately half of male infertility cases, sperm dysfunction is probably due to the sequence of elevated ROS levels [8].

As seminal parameters are directly associated with serum lipids and SDF, tracing a direct correlation between serum lipids and SDF is possible. Serum lipid profile, being the most commonly performed investigation, can be utilized as a marker for possible SDF. It can be used to evaluate male infertility before the application of artificial reproduction techniques (ART) if a correlation is detected between serum lipid and SDF.

In this novel study, we aim to investigate the prevalence of SDF among men with abnormal semen parameters and explore potential correlations between SDF and serum lipid values. By examining this previously unexplored relationship, we seek to enhance our understanding of the multifaceted factors influencing male fertility and contribute valuable insights that may inform future diagnostic and therapeutic approaches to managing male infertility.

## Materials and methods

This hospital-based cross-sectional study was conducted in the Department of Anatomy in collaboration with the Department of Obstetrics and Gynaecology and the Department of Biochemistry at Jawaharlal Institute of Postgraduate Medical Education and Research (JIPMER), Puducherry, India from July 2018 to June 2020. The study was approved by the Institutional Ethics Committee (approval no. JIP/IEC/2018/0156) as per the Declaration of Helsinki. The study participants were briefed about the study protocol before enrolling and gave their written informed consent.

The sample size required for the study is 106, estimated using the statistical formula for the correlation coefficient. The minimum expected sample correlation is 0.65, with a population correlation of 0.50 SDF with serum lipid profile [4,9]. The sample is estimated at 5% significance and 90% power.

We recruited consecutive eligible individuals with abnormal semen analysis for the study. We enrolled 125 patients who attended the infertility clinic at JIPMER between June 2018 and June 2020. Out of these, 15 patients failed to give fasting blood samples for lipid profile, and four patients who were diagnosed with diabetes later were excluded from the study. Thus, a total of 106 infertile males with abnormal semen analysis per the WHO criteria (2010), were included in the study. The inclusion criteria were all male partners attending the infertility clinic at the Department of Obstetrics and Gynaecology and aged between 21 and 50 years with abnormal semen parameters. The exclusion criteria included reproductive organ disorders such as undescended testis, prostatitis, urinary tract infections, testicular carcinoma, and a history of chronic diseases such as diabetes mellitus, hypertension, hypothyroidism, etc., that lead to dyspermia.

Semen analysis

After sexual abstinence for two to three days, semen samples were collected in sterilized containers from the participants. Specimens were kept at room temperature for 30 to 40 minutes, enabling the semen to liquefy. After semen liquefaction, conventional semen analysis was done in the Central Pathology Laboratory, using the SQA-V Gold Automated Semen Analyzer (Medical Electronic Systems, Encino, CA, USA). The analysis was done according to the WHO (2010) guidelines. Semen samples, after analysis, were stored at -80◦C (119) (New Brunswick Scientific Ultra-Low Temperature Freezer, Eppendorf Inc., Framingham, MA, USA) in the Department of Anatomy. Sperm parameters were considered abnormal if sperm concentration was <15 x106/ml, sperm total motility was <40% and sperm morphology was <4%.

Comet assay procedure

Sperm DNA fragmentation was demonstrated in sperm cells using single-cell gel electrophoresis (comet assay). Comet assay is the simplest, easiest, and fastest method for measuring DNA damage in mammalian cells. On the day of the procedure, the frozen sample was thawed by gradually bringing it to room temperature, and the following procedure laid out by Singh et al. in 2003, was carried out [10].

Procedure for Optimisation of Sperm Cells

The semen sample was diluted with phosphate-buffered saline (PBS) in a clean and dry centrifuge tube (15ml) to achieve the final sperm concentration of 5 to 6 x106 per ml. Dilution was based on the sperm concentration in the semen, which had already been measured using the SQA-V Gold Automated Semen Analyzer.

Preparation of Slides

Pre-coating of agarose: Over one end of the slide, aspirated 50µl of hot 0.7% high-resolution agarose (HRA) was dropped with the help of a micropipette. It was then smeared in the opposite direction with the help of another plain slide inclined at about an angle of 45^o^. Slides were kept horizontal on a flat surface for drying.

Coating of agarose: An aspirated 200µl of hot 1% HRA with a micropipette was placed in the middle of the pre-coated slide. A coverslip was placed carefully over the gel to form a uniform layer over the pre-coated slide, avoiding air bubble formation. The agarose was solidified at room temperature for five minutes, and the coverslip was removed gently.

Layering of the sperm-HRA gel mixture: In a microcentrifuge tube, 50μl of 0.7% HRA (37^o^C) was added to 10μl of PBS-diluted sperm and was mixed thoroughly by pipetting. The cell agarose mixture was dropped over the HRA layer. A coverslip was placed carefully over the cell agarose layer, avoiding air bubble formation. The cell agarose mixture was solidified at room temperature for five minutes, and the coverslip was removed gently.

Layering of the fourth layer of gel: A pipetted 200μl of 0.7% HRA was placed over the cell agarose mixture, and a fresh coverslip was placed carefully to avoid air bubbles. The agarose layer was solidified at room temperature for five minutes. Following this, the coverslip was gently removed.

Procedure for Lysis of Sperm

The slides were gently immersed into a Coplin jar containing prewarmed lysing solution and incubated at 37^o^C for two hours.

Procedure for Electrophoresis of Slides

The slides were gently removed from the lysis solution and placed perpendicular to both electrodes, with the agarose-coated side facing upwards in the horizontal submarine gel electrophoresis system. The electrophoresis tank was filled with fresh, cold electrophoresis buffer until the buffer completely covered the slides without forming air bubbles over the agarose gel. Cold electrophoresis buffer was used to avoid DNA damage due to heat generated during the flow of current. The slides were kept in the electrophoresis buffer for 30 minutes for equilibration. After equilibration, electrophoresis was carried out for 30 minutes at 12V and 250mA.

Procedure for Neutralisation

The slides were then gently lifted from the electrophoresis buffer and placed in the Coplin jar. The slides were carefully flooded with neutralizing buffer and removed after 30 minutes. The process was repeated twice. After neutralization, the slides were kept on an aluminum tray and air-dried overnight at room temperature.

Procedure for Staining

A clean coverslip was used to cover the slide after dropping 100µl of ethidium bromide stain over the slide. Before viewing the slides, excess stains from the back and edges of the slides were blotted away. Ethidium bromide was cautiously handled because of its carcinogenic properties. Olympus BX53 Fluorescent Microscope (Evident Corp., Tokyo, Japan) equipped with an excitation filter of 515nm to 560nm and a barrier filter of 590nm was used to visualize the ethidium bromide-stained slides. The images were captured for comet scoring under 200X magnification. Slides were analyzed immediately since the slides stained with ethidium bromide cannot be stored.

Comet Scoring

The DNA damage was estimated by measuring the length of the comet tail and visual scoring of the degree of damage from 0 to 4 according to the comet's appearance. The captured images were converted into .bmp files using easy-to-convert jpeg to bitmap (BMP) software available online. The converted images were analyzed using TriTek Comet ScoreTM Freeware version 1.5 (Tritek Technologies Inc., Wilmington, DE, USA). The software was used to quantify DNA damage parameters such as comet length, head diameter, percentage of DNA in the head, tail length, and percentage of DNA in the tail. In total, 40 to 50 randomly selected cells were analyzed per sample. Comets were selected without bias such that the comets represented the entire sample. Comets seen in the edges, air bubbles, and overlapped images were rejected.

Estimation of serum lipid parameters

The fasting serum samples were analyzed for the levels of serum total cholesterol (TC), triglycerides (TG), low-density lipoprotein (LDL), high-density lipoprotein (HDL), and very low-density lipoprotein (VLDL). The serum lipid profile was analyzed in Beckman Coulter AU5800 Chemistry Autoanalyzer (Beckman Coulter Inc., Brea, CA, USA) by spectrophotometric assay. Serum TC was estimated by the cholesterol oxidase-peroxidase method; serum TG was estimated with the lipase/glycerol phosphate oxidase-peroxidase method; serum LDL and HDL were estimated via the cholesterol esterase-cholesterol oxidase-peroxidase method. Serum VLDL was calculated using Friedwald's formula, i.e., VLDL = triglyceride (mg/dl)/5. The standardized methodology and equipment used to estimate lipid parameters in our samples have proven the reliability of lipid profile measurements. The LDL-cholesterol (LDL-C) has been directly measured, and not calculated using the commonly employed Friedewald's equation. The LDL-C has been estimated using the cholesterol esterase-cholesterol oxidase-peroxidase method. Moreover, only fasting samples were collected for lipid profiles to avoid diet interference in triglycerides estimation.

Statistical analysis

Continuous variables were expressed as mean and standard deviation or median and interquartile ranges (IQR) based on the normality of data (Kolmogorov-Smirnov test). The correlation between the variables was calculated by Pearson's correlation between normal distribution variables and Spearman's rho correlation between non-normal distribution variables. A p-value <0.05 was considered statistically significant. All statistical analyses were done using SPSS Statistics version 23.0 (IBM Corp., Armonk, NY, USA).

## Results

We recruited 106 males with abnormal semen parameters based on the inclusion and exclusion criteria. The age of most of the participants with male infertility was between 26 and 35 years. Out of the 106 infertile men, 52% (n=55) had SDF, i.e., a total comet length of more than 51 ± 12.69 µm (Table 1).

**Table 1:** Age-wise comparison of total comet length

Age group (n = 106)	Total comet length (µm) (Mean ± SD)
21 to 25 (n = 12)	48.63 ± 13.97
26 to 30 (n = 32)	52.62 ± 11.47
31 to 35 (n = 39)	49.51 ± 13.92
36 to 40 (n = 16)	51.64 ± 12.38
41 to 45 (n = 7)	54.46 ± 10.71

Sperm total motility and sperm morphology were found to be decreased in the infertile males (Table 2).

**Table 2:** Distribution of semen parameters in infertile males

Semen parameters			Lower reference limit
Sperm total motility (%)	Mean ± SD	22 ± 2.66	40%
Sperm morphology (%)	Median (Range)	2 (0 – 13)	4%
Sperm concentration (x 10^6^/ml)		39 (1 – 200)	15 x 10^6^/ml

Serum TG, LDL, and VLDL were high in the infertile males (Table 3).

**Table 3:** Distribution of serum lipid values in infertile males TC: Total cholesterol, HDL: High-density lipoprotein, LDL: Low-density lipoprotein, TG: Triglycerides, VLDL: Very low-density lipoprotein

Serum lipid values			Reference value
TC (mg/dl)	Mean ± SD	180.30 ± 37.90	<200 mg/dl
HDL (mg/dl)		41.60 ± 8.93	>40 mg/dl
LDL (mg/dl)		111.26 ± 24.65	<100 mg/dl
TG (mg/dl)	Median (Range)	152 (82 – 382)	<150 mg/dl
VLDL (mg/dl)		30 (16 – 76)	<30 mg/dl

The mean comet length observed in the study participants was 51 ± 12.69 (Table 4).

**Table 4:** Distribution of comet parameters in infertile males

Comet parameters	Mean ± SD
Total length (µm)	51 ± 12.69
Head diameter (µm)	26.30 ± 5.35
Percentage of DNA in head	68.94 ± 12.53
Tail length (µm)	24.78 ± 11.56
Percentage of DNA in Tail	31.06 ± 12.53

A correlation analysis was performed between each semen parameter and comet parameters, which showed no significant correlation (Table 5).

**Table 5:** Correlation between semen and comet parameters

Semen parameter	Comet parameter	Correlation coefficient r	p-value
Sperm total motility	Total comet length	-0.101	0.302
	Head diameter	-0.123	0.207
	Percentage of DNA in comet head	-0.014	0.889
	Tail length	-0.051	0.601
	Percentage of DNA in comet tail	0.014	0.889
Sperm morphology	Total comet length	0.034	0.730
	Head diameter	-0.065	0.511
	Percentage of DNA in comet head	-0.051	0.604
	Tail length	0.099	0.312
	Percentage of DNA in comet tail	0.051	0.604
Sperm concentration	Total comet length	-0.016	0.874
	Head diameter	0.102	0.297
	Percentage of DNA in comet head	-0.010	0.918
	Tail length	-0.064	0.514
	Percentage of DNA in comet tail	0.010	0.918

A correlation analysis was performed between lipid profile and semen parameters, which showed no significant correlation (Table 6).

**Table 6:** Correlation between serum lipid profile and semen parameters TG: Triglycerides, HDL: High-density lipoprotein, LDL: Low-density lipoprotein, VLDL: Very low-density lipoprotein

Serum lipid profile	Semen parameter	Correlation coefficient r	p-value
Serum cholesterol	Sperm total motility	-0.092	0.347
	Sperm morphology	-0.109	0.265
	Sperm concentration	0.148	0.131
Serum TG	Sperm total motility	-0.061	0.532
	Sperm morphology	-0.026	0.789
	Sperm concentration	0.103	0.294
Serum HDL	Sperm total motility	0.052	0.598
	Sperm morphology	0.047	0.633
	Sperm concentration	0.147	0.132
Serum LDL	Sperm total motility	-0.099	0.312
	Sperm morphology	-0.077	0.430
	Sperm concentration	0.063	0.523
Serum VLDL	Sperm total motility	-0.048	0.624
	Sperm morphology	-0.024	0.807
	Sperm concentration	0.100	0.306

A correlation analysis was performed between lipid profile parameters with comet parameters, which shows that serum TG, serum LDL, and serum VLDL show a weak and negative correlation with DNA in the comet head and a weak positive correlation with the DNA percentage in the tail (Table 7).

**Table 7:** Correlation between serum lipids and comet parameters *A p-value <0.05 is considered statistically significant. TG: Triglycerides, HDL: High-density lipoprotein, LDL: Low-density lipoprotein, VLDL: Very low-density lipoprotein

Serum lipid parameter	Comet parameter	Correlation coefficient r	p-value
Serum cholesterol	Total comet length	0.043	0.664
	Head diameter	0.005	0.963
	Percentage of DNA in comet head	-0.156	0.111
	Tail length	0.044	0.655
	Percentage of DNA in comet tail	0.156	0.111
Serum TG	Total comet length	0.113	0.247
	Head diameter	-0.090	0.360
	Percentage of DNA in comet head	-0.213	0.028^*^
	Tail length	0.159	0.103
	Percentage of DNA in comet tail	0.213	0.028^*^
Serum HDL	Total comet length	-0.053	0.588
	Head diameter	0.051	0.602
	Percentage of DNA in comet head	0.032	0.743
	Tail length	-0.080	0.413
	Percentage of DNA in comet tail	-0.032	0.743
Serum LDL	Total comet length	0.104	0.289
	Head diameter	0.030	0.762
	Percentage of DNA in comet head	-0.222	0.022^*^
	Tail length	0.099	0.312
	Percentage of DNA in comet tail	0.222	0.022^*^
Serum VLDL	Total comet length	0.098	0.316
	Head diameter	-0.101	0.301
	Percentage of DNA in comet head	-0.215	0.027^*^
	Tail length	0.148	0.130
	Percentage of DNA in comet tail	0.215	0.027^*^

## Discussion

We proposed this study to determine the proportion of SDF and the correlation of SDF with serum cholesterol, TG, HDL, LDL, and VLDL in males with abnormal semen parameters. Studies have shown the association between semen parameters and serum lipid levels [7,9,11,12] and the association between sperm DNA damage and semen parameters [10,13-15].

The extent of sperm DNA damage may act as an indicator of the outcome of natural conception and in-vitro fertilization (IVF). It can be an essential adjuvant to routine semen analysis. In this study, we investigated the correlation between SDF and semen parameters. We could not observe a significant correlation between any semen parameters and SDF. The observation was contrary to most previous studies reporting a significant association between the two. Among the previously reported studies, a prospective study showed that sperm DNA damage was positively associated with semen volume and sperm morphology and negatively associated with sperm motility and sperm concentration [10]. Also, a study reported an inverse correlation between SDF and semen parameters (sperm motility, sperm morphology, and sperm concentration) [14].

Many researchers have explored the relationship between serum lipids and human fertility, as cholesterol is the primary source of steroid synthesis and has a decisive role in spermatogenesis [16]. The link between the two can be explained based on the lipid peroxidation reaction. The pathological chain reaction in which the free radicals acquire a single electron from membrane phospholipids and, as a result, an unpaired electron is left behind in the membrane phospholipid, leading to ROS or free radical formation as metabolism products, is termed lipid peroxidation [17,18]. Polyunsaturated fatty acid (PUFA) in the cellular membrane gets damaged due to oxidative stress. It leads to the release of end products of lipid peroxidation, which serve as free radicals in excess [19]. Lipids in the plasma membrane of sperm, such as the PUFA, are the most susceptible to oxidative stress [20]. Damage to the plasma membrane leads to impaired sperm function, such as decreased motility and failure to undergo sperm-oocyte fusion. Sperm functions are impaired due to damage to the plasma membrane of the sperm. Thus, a reduction in sperm motility and sperm-oocyte fusion failure are noted [21,22]. Similarly, there is a reduction in sperm motility in our study, but it is non-significant. Lucjaz et al. state that lipid peroxidation products can react with DNA, leading to genotoxicity [23].

Considering the relation between semen parameters and serum lipid values, semen parameters, and SDF, we explored a possible link between SDF and serum lipid values. The studies focusing on the association between semen quality and lipid profiles have reported diverse conclusions. Schisterman et al. showed that higher serum cholesterol levels and serum phospholipids were significantly associated with a lower percentage of sperm with a small head and intact acrosome (sperm morphology) [11]. Hagiuda et al. revealed no significant association between sperm concentration or sperm motility and serum TG. Still, a positive association was observed between sperm morphology and serum TG [12]. Luet al. showed no significant correlation between semen parameters and serum lipid profiles [7]. Liu et al. showed that sperm concentration was positively associated with serum TG and VLDL. There was a correlation between serum TC and increasing total sperm motility. The serum LDL levels were positively related to total and progressive sperm motility [9]. In a similar study, Lu et al. reported no significant correlation between SDF and any serum lipid values [10]. Our study observed a modest positive correlation between the percentage of DNA in the comet tail and serum lipids (serum TG, VLDL, and LDL) and an inverse correlation with the percentage of DNA in the comet head.

Limitations of the study

The conflicting results, i.e., the lack of association between semen parameters and SDF, might be justified by the difference in the study population, sperm preparation methods, and inherent characteristics of the technique of DNA fragmentation. The correlation noted in our study would have had additional validation if the average comet score (average SDF across 100 individual spermatozoa), high comet score (the proportion of spermatozoa with high DNA damage), and low comet score (the proportion of spermatozoa with low DNA damage) were considered. The nutritional status of the study participants has not been taken into consideration, which can be a potential confounder for the study results. Hence, large-scale studies are needed to confirm the role of lipids and lipoproteins in predicting male infertility.

Implications of the study

Our study summarizes the significance of evaluating lipid parameters as one of the profiles in investigating male infertility. The findings of a modest correlation between SDF and serum lipid parameters in this study pave the way for future explorations targeting lipid profiles, thus simplifying the complexity surrounding male infertility, which can be supported and validated by future studies on this topic. Our research has proven a modest association between serum lipid parameters and SDF. Thus, in routine clinical practice, after establishing cut-off values and standardized algorithms using simple, routine lipid parameters, it is valid to perform SDF testing for unexplained male infertility with altered lipid parameters before considering ART.

## Conclusions

The current study is novel in terms of hypothesis and population or region. A notable prevalence of 52% of men with abnormal semen parameters had an SDF of more than 51 ± 12.69 (μm) as determined by the comet assay. A weak positive and negative correlation was observed between SDF regarding the percentage of DNA in the head and tail and serum lipid values. There were also some instances where no correlation was observed between SDF and serum lipid values. Ours is the only study that noted a modest correlation between serum lipids and SDF, which serves as a seed for further follow-up studies.
